# Metabolic Checkpoints in Rheumatoid Arthritis

**DOI:** 10.3389/fphys.2020.00347

**Published:** 2020-04-17

**Authors:** Valentina Pucino, Michelangelo Certo, Gilda Varricchi, Giancarlo Marone, Francesco Ursini, Francesca Wanda Rossi, Amato De Paulis, Claudio Mauro, Karim Raza, Christopher Dominic Buckley

**Affiliations:** ^1^Institute of Inflammation and Ageing, College of Medical and Dental Sciences, University of Birmingham, Birmingham, United Kingdom; ^2^Rheumatology Research Group, Institute for Inflammation and Ageing, College of Medical and Dental Sciences, University of Birmingham, Queen Elizabeth Hospital, Birmingham, United Kingdom; ^3^Department of Translational Medical Sciences (DiSMeT) and Center for Basic and Clinical Immunology Research (CISI), University of Naples Federico II, Naples, Italy; ^4^Department of Public Health, University of Naples Federico II, Naples, Italy; ^5^Ospedale dei Colli, Hospital Pharmacy, Naples, Italy; ^6^Section of Rheumatology, Department of Biomedical and Neuromotor Sciences (DiBiNeM), University of Bologna, Bologna, Italy; ^7^Medicine and Rheumatology Unit, IRCCS Istituto Ortopedico Rizzoli, Bologna, Italy; ^8^Institute of Cardiovascular Sciences, College of Medical and Dental Sciences, University of Birmingham, Birmingham, United Kingdom; ^9^Institute of Metabolism and Systems Research, College of Medical and Dental Sciences, University of Birmingham, Birmingham, United Kingdom; ^10^Research into Inflammatory Arthritis Centre Versus Arthritis, College of Medical and Dental Sciences, University of Birmingham, Queen Elizabeth Hospital, Birmingham, United Kingdom; ^11^MRC and Versus Arthritis Centre for Musculoskeletal Ageing Research (CMAR), College of Medical and Dental Sciences, University of Birmingham, Queen Elizabeth Hospital, Birmingham, United Kingdom; ^12^The Kennedy Institute of Rheumatology, University of Oxford, Oxford, United Kingdom

**Keywords:** rheumatoid arthritis, metabolism, immunity, mediators of inflammation, immunometabolism

## Abstract

Several studies have highlighted the interplay between metabolism, immunity and inflammation. Both tissue resident and infiltrating immune cells play a major role in the inflammatory process of rheumatoid arthritis (RA) via the production of cytokines, adipo-cytokines and metabolic intermediates. These functions are metabolically demanding and require the most efficient use of bioenergetic pathways. The synovial membrane is the primary site of inflammation in RA and exhibits distinctive histological patterns characterized by different metabolism, prognosis and response to treatment. In the RA synovium, the high energy demand by stromal and infiltrating immune cells, causes the accumulation of metabolites, and adipo-cytokines, which carry out signaling functions, as well as activating transcription factors which act as metabolic sensors. These events drive immune and joint-resident cells to acquire pro-inflammatory effector functions which in turn perpetuate chronic inflammation. Whether metabolic changes are a consequence of the disease or one of the causes of RA pathogenesis is still under investigation. This review covers our current knowledge of cell metabolism in RA. Understanding the intricate interactions between metabolic pathways and the inflammatory and immune responses will provide more awareness of the mechanisms underlying RA pathogenesis and will identify novel therapeutic options to treat this disease.

## Introduction

Rheumatoid arthritis is an immune mediated inflammatory disease characterized by autoantibody production [including rheumatoid factor (RF) and anti–citrullinated protein antibody (ACPA), anti-carbamylated proteins antibodies (anti-CarP) etc.,] chronic synovial inflammation (synovitis) and hyperplasia, cartilage and bone destruction, as well as systemic complications such as cardiovascular, pulmonary, and neurological co-morbidity. Progressive disability and systemic complications are still a burden leading to socioeconomic costs and unmet needs. Indeed, current conventional and biologic disease modifying therapies produce good responses in only 60% of patients (Humby et al., 2019). Predictive biomarkers of prognosis, therapeutic response, and resistance to treatment, which currently include ACPA, RF, C-reactive protein (CRP), and erythrocyte sedimentation rate (ESR), remain inadequate from a clinical decision making perspective (McInnes and Schett, 2011; Dennis et al., 2014).

**FIGURE 1:**
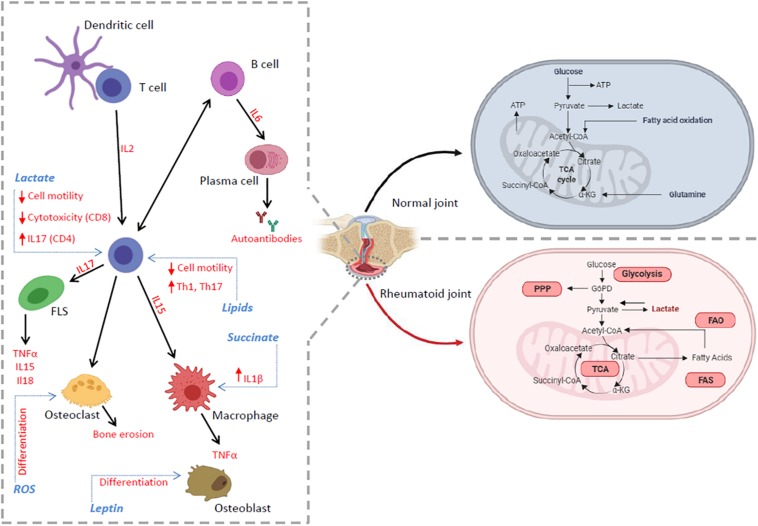
The inflammatory environment in RA synovium. The RA tissue microenvironment is characterized by the accumulation of cytokines, adipo-cytokines and metabolic intermediates produced by the accelerated metabolism of infiltrating and tissue resident immune cells. These events can promote or be the consequence of a dysregulation in several metabolic pathways (red), including glycolysis, TCA cycle, PPP and lipid metabolism, which regulate many cell functions including activation, differentiation, proliferation, autoantibody and cytokine production leading to pro-inflammatory immune responses and the exacerbation of chronic inflammation.

The loss of immune tolerance that precedes the onset of inflammation in the joint is thought to represent a key process in RA pathogenesis (McInnes and Schett, 2011; Smolen et al., 2016) and is likely to occur at extra-articular sites (Tracy et al., 2017). Synovitis, the hallmark of established RA, is characterized by leukocyte infiltration, neo-angiogenesis and increased expression of adhesion molecules and chemokines which lead to increased leukocyte migration into the inflamed site. In addition, inadequate lymphangiogenesis, which limits cell egress, together with local fibroblast activation, promotes the establishment of synovial inflammation (Croft et al., 2019). Nutrient availability is also limited and immune and joint resident cells compete for available nutrients at a rate which exceeds their production thereby increasing the metabolic demand (Figure 1; Goetzl et al., 1971; Treuhaft and MCCarty, 1971; Patella et al., 2015; Biniecka et al., 2016; Tsokos, 2016; Yang et al., 2016; Zhou et al., 2016). All these events can, in the long-term, induce an alteration of immune responses and promote a continued breach of immune tolerance leading to inflammation and autoimmunity.

## Metabolites: A Focus on Lactate

The study of intermediates and end-products of metabolism in the context of immune cell functions is an emerging field that has been termed immunometabolism (Pearce et al., 2013). It is now clear that molecules such as succinate, lactate, acetyl-CoA, fumarate are more than intermediate by-products in metabolic pathways as they function as signaling molecules capable of linking metabolic reprograming with immune and inflammatory responses in immunity, inflammation and cancer (Figure 1; Haas et al., 2016). Whether metabolic perturbations are causal or the effect of the disease and how they can impact on the prognosis of RA is an area of significant current research.

Nuclear magnetic resonance (NMR) spectroscopy–based metabolomics on serum and urine samples from people with RA has identified a metabolic signature of patients with active established RA which differs from that of healthy controls (Young et al., 2013). Among the metabolites investigated 3-hydroxybutyrate and lactate were much higher in RA than in the control group. In addition choline, lactate and low-density lipoprotein (LDL) lipids strongly correlated with CRP a marker of disease activity (Young et al., 2013). This evidence suggests that NMR could be used as a tool to predict the development of atherosclerosis and other metabolic complications often associated with inflammatory disease. Similarly, a gas chromatography–mass spectrometry (GC–MS) study on serum samples, has shown a decrease in amino acid and glucose metabolism in combination with increased fatty acid metabolites such as palmitate, oleate and cholesterol (Zhou et al., 2016).

In the same vein, a correlation between serum metabolites and gene expression profiling in synovial tissue from patients with active RA was recently found (Narasimhan et al., 2018). The authors described an association of serine, glycine, and phenylalanine metabolism with a lymphoid cell gene expression signature in synovial tissue. In addition, amino acids (i.e., alanine, aspartate, glutamate) and choline-derived metabolites correlated with TNF-α synovial expression while circulating ketone bodies associated with synovial gene expression of metalloproteinases. These data pointed to a link between serum metabolite profiles and synovial biomarkers further suggesting that NMR may be a promising technique for mapping pathogenic pathways in RA (Narasimhan et al., 2018).

*In vitro* studies have further highlighted the role of metabolites as signaling molecules in mediating inflammatory responses. Studies on succinate have shown that lipopolysaccharides (LPS)-activated inflammatory (M1) macrophages accumulate this metabolite intracellularly as a consequence of an altered TCA cycle (Jha et al., 2015). Here succinate promotes the activation of hypoxia-inducible factor (HIF)-1α and increases pro-inflammatory interleukin (IL)-1β production. In addition, when activated by inflammatory stimuli, macrophages release succinate into the extracellular space and up-regulate G protein-coupled receptor (GPR)91, which functions as a sensor for extracellular succinate to enhance IL-1β production (Tannahill et al., 2013). Notably, GPR91-deficient mice display decreased macrophage activation and reduced IL-1β production during antigen-induced arthritis as well as decreased dendritic cell traffic and reduced differentiation of Th17 cells in the lymph nodes (Tannahill et al., 2013; Saraiva et al., 2018). High levels of succinate have been found in synovial fluid from RA patients, where it induces IL-1β release from macrophages in a GPR91-dependent manner. This evidence suggests that GPR91 antagonists may act as novel therapeutic molecules to treat RA (Littlewood-Evans et al., 2016). Interestingly, intracellular and extracellular succinate exhibit different functions. More specifically, intracellular succinate induces angiogenesis through HIF-1α, while extracellular succinate regulates GPR91 activation (Li et al., 2018). The abolition of succinate dehydrogenase (SDH) activity with dimethyl malonate limited succinate accumulation and prevented angiogenesis via blocking the HIF-1α/VEGF axis, revealing a new potential therapeutic strategy to attenuate neo-angiogenesis in arthritis (Li et al., 2018). If succinate exhibits pro-inflammatory activity, other metabolites such as fumarate and itaconate, have been observed to mediate anti-inflammatory effects (McGuire et al., 2016; Mills et al., 2018). With regard to fumarate, the methyl ester dimethyl fumarate (DMF) has been approved for the treatment of relapsing multiple sclerosis (MS) (Fox et al., 2012; Gold et al., 2012). Interestingly DMF has been reported to reduce osteoclastogenesis and bone destruction *via* increasing the expression of nuclear factor erythroid 2–related factor 2 (NRF2)-mediated antioxidant genes and decreasing reactive oxygen species (ROS) levels (Yamaguchi et al., 2018). The role of itaconate in RA is still debated. Despite evidence suggests an anti-inflammatory role (Mills et al., 2018) other studies have shown that reduced levels of itaconate correlate with a decreased pro-inflammatory (M1) signature in human macrophages isolated from healthy control subjects (Papathanassiu et al., 2017) and with a reduced arthritis severity *in vivo* (Michopoulos et al., 2016; Papathanassiu et al., 2017). It would be valuable to investigate how these observations in murine models translate into the human disease setting (i.e., OA vs. RA macrophages).

For more than 50 years, the inflamed joint has been recognized as a site with low levels of glucose and high amounts of lactate (Goetzl et al., 1971; Treuhaft and MCCarty, 1971), as a consequence of the intense cellular turnover in the synovium. Accumulation of lactate in RA synovial fluid is in part responsible for the acidic environment of RA synovitis. Indeed, it is well established that the PH of synovial fluidis significantly lower in inflamed arthritic joints than in healthy joints (Cummings and Nordby, 1966).

The rheumatoid synovial environment is paradigmatic of some of the lactate-induced features seen in T cells, including IL-17 secretion and loss of antigen responsiveness (Croia et al., 2013). In particular, lactate modulates specific T cell subsets via the interaction with lactate transporters. Sodium lactate selectively affects CD4^+^ T cell functions via the solute carrier (SLC)5A12, while lactic acid was found to have an impact on CD8^+^ T cell motility and cytolytic capability via its influx through SLC16A1 (MCT1) (Haas et al., 2015; Pucino et al., 2017).

Solute carrier 5A12 is highly expressed in RA synovial tissues and this expression significantly increases in association with the inflammatory T cell score (Haas et al., 2015; Pucino et al., 2017). Notably we showed that SLC5A12 blockade promoted the egress of CD4^+^ T cell from the inflamed tissue in an organ culture model and improved clinical scores of disease in an experimental model of arthritis (Pucino et al., 2019).

Another lactate transporter, the monocarboxylate transporter 4 (MCT4 or SLC16A3) was found to be up-regulated by RA synovial fibroblast (FLS) compared to osteoarthritis (OA) FLS (Fujii et al., 2015). Silencing MCT4, with MCT4-specific siRNA, inhibited the proliferation of RA FLS and was able to reduce the severity of arthritis in mice with collagen-induced arthritis (CIA) (Fujii et al., 2015).

These findings have established lactate signaling as integral feature of RA and open up the possibility of a new biomarker for disease progression and response to treatment as well as a novel target for therapeutic intervention. However, a better understanding of how the different synovial cell types co-ordinate their metabolism and the role of metabolites in cell-cell communication will be required to fully appreciate how the metabolic landscape in disease differs from that in health.

## Glucose Metabolism

Proliferating cells mainly use aerobic glycolysis (Warburg effect) to generate energy. Indeed, in inflammatory conditions and tumors, aerobic glycolysis is preferred over oxidative phosphorylation for the production of ATP and for the stock of carbon sources necessary to build cell mass (Tsokos, 2016).

Both peripheral and tissue resident RA CD4^+^ T cells have a unique metabolic signature (Weyand et al., 2017; Pucino et al., 2019). Indeed, RA CD4^+^ T cells exhibit an impairment in engaging glycolysis. This is due to a deficiency of 6-phosphofructo-2-kinase/fructose-2,6-bisphosphatase (PFKFB3), a glycolysis regulator enzyme, resulting in delayed glycolysis and increased pentose phosphate pathway (PPP) via the up-regulation of glucose-6-phosphate dehydrogenase (G6PD). As a consequence, high levels of NADPH (reduced form nicotinamide adenine dinucleotide phosphate) and ROS consumption were observed. Moreover, altered activation of ataxia telangiectasia mutated (ATM), an enzyme involved in the cell cycle, was also reported. All these alterations result in increased cellular proliferation, a switch toward pro-inflammatory CD4^+^ T cell subsets (Th1 and Th17) and chronic inflammation (Yang et al., 2013, 2016). Interestingly the replenishment of ROS was able to reverse these phenomena (Yang et al., 2013, 2016). Similarly, CD4^+^ T cell from naive-to-treatment RA synovial tissues display a reduced expression of glycolytic genes coupled with increased PPP and Kreb cycle genes (Pucino et al., 2019). These findings correlate with increased Th17 cell tissue infiltration and the formation of ectopic lymphoid structure (ELS) (Pucino et al., 2019).

6-phosphofructo-2-kinase/fructose-2 deficiency also limits the ability of RA T cells to engage autophagy with increased susceptibility to apoptosis (Yang et al., 2013). This is linked with the recent discovery that RA T cells lack N-myristoyltransferase (NMT)-induced AMP-activated protein kinase (AMPK) activation which is a positive regulator of autophagy by suppressing the mammalian target of rapamycin (mTOR) activity (Kim et al., 2013; Cassano et al., 2014; Wen et al., 2019). Further studies are needed to better comprehend the intracellular mechanisms linking metabolism, apoptosis, and autophagy in RA to understand potential therapeutic implications.

In contrast to T cells, RA FLS display increased glycolytic metabolism under metabolic stress (Falconer et al., 2018). Indeed, glucose deprivation or glycolytic inhibitors [i.e., 2-deoxy-D-glucose (2-DG)], reduced FLS cytokine secretion, proliferation, and migration as well as disease severity in a mouse model of arthritis (Garcia-Carbonell et al., 2016). In this context, RA FLS show an higher expression of the inducible isoform of hexokinase (HK)2, which catalyze the phosphorylation of glucose to glucose 6 phosphate (G6P), in comparison to OA FLS. Interestingly, HK2 silencing reduced RA FLS tissue invasiveness; by contrast, the overexpression of HK2 increased the levels of MMP, IL6, and IL8 along their migratory rate (Bustamante et al., 2018). These data were further confirmed *in vivo*, in a mouse model of arthritis, where the HK2 deletion in murine FLS ameliorated disease severity of arthritis (Bustamante et al., 2018). Similarly, the HK2 inhibitor, 3-bromopyruvate (BrPA), was found to modulate the Th17/Treg ratio and suppress dendritic cells (DC) activation and cytokine expression (Okano et al., 2017). In addition to its canonical role in glucose metabolism, HK2 translocates to mitochondria where it triggers an autophagic and anti-apoptotic responses through its interaction with the voltage-dependent anion channel (VDAC) (Tan and Miyamoto, 2015). Intriguingly we found that lactate, which is abundant in the RA synovium, modulates HK2 mitochondrial translocation suggesting a potential role of this enzyme in promoting T cell survival. This provides an important link between metabolism and apoptosis resistance in the RA synovium that needs to be further explored (Pucino et al., 2019).

Abnormal metabolism by RA FLS may be a consequence of the hypoxic microenvironment found in inflamed sites. Indeed, hypoxia by itself is able to induce a downregulation of mitochondrial respiration and an increase of glycolysis in RA fibroblasts, leading to synovial invasiveness, angiogenesis and synovial hyperplasia (Biniecka et al., 2014, 2016). Moreover stimulation *in vitro* of RA FLS with platelet derived growth factor (PDGF) or TNF increased glucose metabolism (Garcia-Carbonell et al., 2016).

Enhanced glycolysis is also observed in synovial monocytes and macrophages in RA. RA macrophages express high levels of the glycolytic enzyme α-enolase, which induces secretion of pro-inflammatory cytokines through autoantibody recognition (Bae et al., 2012). High concentrations of glucose have also been shown to increase IL-1β secretion from RA monocytes through an NOD-, LRR- and pyrin domain-containing protein 3 (NLRP3)/inflammasome-dependent mechanism (Ruscitti et al., 2015) and the glycolytic enzyme HK1 is known to drive cleavage and activation of pro–IL-1β in macrophages (Moon et al., 2015). Following these results, a clinical trial (NCT02236481) has recently been published showing the efficacy of IL-1 inhibition, in terms of RA disease activity and glycated hemoglobin percentage (HbA1c%), as a targeted treatment in patients with RA and type 2 diabetes (T2D). Notably patients treated with TNF inhibitors did not achieve the same results (HbA1c reduction) in this trial suggesting a different pathogenic mechanism linking inflammation, T2D and RA. Further studies are needed to dissect the implication of NLRP3 and the risk of developing T2D in patients with RA (Ruscitti et al., 2019) and to highlight the potential application of NLRP3-targeted therapies for these diseases. Driven by surrounding environmental conditions, glycolytic enzymes can translocate to the nucleus (“moonlighting”), where they regulate the expression of their target mRNAs and modulate immune responses (De Rosa et al., 2015; Boukouris et al., 2016). For instance, the glycolytic enzyme pyruvate kinase M2 (PKM2) plays a crucial role in the regulation of transcription factors and cytokine production in both coronary artery disease (CAD) and RA macrophages (Shirai et al., 2016; Weyand et al., 2017). Specifically, increased ROS production during inflammation, promotes PKM2 dimerization enabling its nuclear translocation and transcription factor STAT3 phosphorylation, thereby enhancing IL-6 and IL-1β production (Shirai et al., 2016; Weyand et al., 2017). Reducing glycolysis, limiting superoxide production and promoting PKM2 tetramerization, repaired the pro-inflammatory phenotype of CAD macrophages (Shirai et al., 2016). Similarly we found that lactate induces the nuclear translocation of PKM2 in activated CD4^+^ T cells, boosting IL-17 production in a STAT3 dependent manner (Pucino et al., 2019).

Emerging evidence suggests that hypoxia and HIFs play a pivotal role in the regulation of several pathophysiological features of RA including synovitis, angiogenesis, and cartilage destruction (Hua and Dias, 2016). In particular, HIF-1α, a master regulator of glycolysis, is highly expressed by macrophages in the RA synovium, compared to macrophages in OA and healthy control synovium (Hollander et al., 2001) suggesting HIF-1α as novel potential therapeutic target. It will be interesting to determine whether these observations reflect up or down regulation of HIF-1α in the same macrophage subset in RA and OA or alternatively is a reflection of different subsets of macrophages in RA and OA (Croft et al., 2019).

Vascular endothelial growth factor-dependent HIF-1α pathways play a key role in endothelial cell (EC) metabolism. Indeed, in response to growth factor stimulation, such as by vascular endothelial growth factor (VEGF), EC become highly activated, proliferative, and acquire migratory capability (Potente et al., 2011; Varricchi et al., 2018) with increased glycolysis (Yeh et al., 2008; Parra-Bonilla et al., 2010; De Bock et al., 2013). Blockade of the glycolytic enzyme, PFKFB3, inhibited angiogenic tube formation *in vivo* and reduced the secretion of pro-inflammatory/angiogenic mediators in RA FLS and EC suggesting a key role of this glycolytic enzyme in promoting angiogenesis and inflammation (Biniecka et al., 2016). G6PI was also found to be important in the regulation of VEGF secretion from RA FLS (Lu et al., 2017). Indeed, in hypoxic conditions, both G6PI and HIF-1α were increased. This phenomenon was accompanied by enhanced proliferation of RA FLS and angiogenic tube formation of human dermal microvascular endothelial cells (HDMECs) *in vivo*. These events were reversed in G6PI loss-of-function experiments, thus confirming the requirement for G6PI in promoting angiogenesis in RA (Lu et al., 2017).

## Mitochondrial Metabolism

Mitochondrial functions in RA are still under investigation. Mitochondrial DNA (mtDNA) mutations and ROS production were found to be higher in RA compared to OA FLS (Da Sylva et al., 2005). In addition, they correlated with elevated MMP expression and a more invasive phenotype of FLS (Harty et al., 2012). Another study showed that mitochondria in macrophages isolated from the RA synovium, produced more ATP, consumed more oxygen and developed inter-organelle connections with the endoplasmic reticulum, forming mitochondria-associated membranes (MAM). MAMs promote mitochondrial hyperactivity and calcium transport, and induce the inactivation of glycogen synthase kinase 3b (GSK3b). In turn, the inactivation of GSK3b increases the production of the collagenase cathepsin K, a macrophage effector molecule, whose levels correlates with RA clinical disease activity (Zeisbrich et al., 2018). Lipopolysaccharide (LPS) stimulated macrophages (M1 macrophages) display a decreased TCA cycle. Moreover the mitochondrial oxidative phosphorylation pathway is coupled to the up-regulation of glucose transporter 1 (Glut1) to facilitate efficient uptake of glucose (Corcoran and O’Neill, 2016). ROS production is increased, partly as a consequence of reversed electron transport in mitochondria, and the accumulation of TCA cycle intermediates such as succinate, as previously described. These events promote the expression of the pro-inflammatory cytokine IL-1β by inhibiting prolyl hydroxylases and activating the transcription factor HIF-1α. Succinate has also been linked to changes in DNA methylation and associated histone proteins which in turn modulate gene expression (Mills and O’Neill, 2014). In animal models of arthritis, succinate has been shown to induce synovial angiogenesis through VEGF-dependent HIF-1α pathways (Li et al., 2018).

In RA, ROS are thought to directly contribute to destructive and proliferative synovitis (Datta et al., 2014). High levels of ROS accumulate in the synovial fluid and peripheral blood of RA patients where they can modify (e.g., via oxidation) major components of cartilage and bone, such as collagen and hyaluronic acid, inducing bone and cartilage destruction (Ishibashi, 2013; Chimenti et al., 2015). Moreover ROS levels positively correlate with disease activity (Datta et al., 2014) and contribute to osteoclast differentiation via RANK signaling (Lee et al., 2005).

## Lipid Metabolism

Recent discoveries have highlighted the role of lipid metabolism in the regulation of immune cells functions (Cucchi et al., 2019) and targeting lipid mediators is becoming an attractive field in autoimmune and allergic disorders (Marone et al., 2019).

It has been recently reported that the short chain fatty acids (FAs) such as acetate, propionate and butyrate are able to orchestrate several CD4^+^ T cell functions by modulating the activity of histone deacetylases (HDAC) (Park et al., 2015) and via the peroxisome proliferator-activated receptor (PPAR) signaling (Berger and Moller, 2002; Klotz et al., 2009; Cipolletta et al., 2012). Lipid metabolism is also crucial for T cell activation and proliferation. Indeed, T cell activation is accompanied by the upregulation of sterol regulatory element binding protein (SREBP). Lack of SREBP by genetic inactivation is detrimental to T cells undergoing clonal expansion after activation (Kidani et al., 2013).

T cells from patients with RA display increased fatty acid synthesis (FAS) leading to their increased tissue invasiveness. More specifically, reduced glycolytic flux due to PFKFB3 deficiency, promotes a shunt toward anabolic glucose utilization (increased PPP and FAS) and the up-regulation of the podosome scaffold adapter protein TKS5 (SH3PXD2A), which is involved in the formation of cell membrane protrusions (Yang et al., 2013; Shen et al., 2017). In addition, enhanced FAS causes the accumulation of cytoplasmic lipid droplets, which are necessary for T cell functions including cell growth, proliferation and for naïve to memory T cell conversion. Interestingly, restoring pyruvate level was able to replenish T cell locomotion and limit tissue-invasiveness and inflammation in non-obese diabetic (NOD) SCID mice (NSG mice) engrafted with human synovial tissue. In addition, inhibition of FAS efficiently reduced tissue inflammation, decreased the number of RANKL^+^ and IFN-γ^+^ T cells and diminished the total number of T cell infiltrating the synovial tissue (Shen et al., 2017). *De novo* FAS regulate Th17 differentiation (Berod et al., 2014). Indeed the inhibition of acetyl-CoA carboxylase (ACC) *in vitro*, using the specific inhibitor Sorafen A, leads to an impaired differentiation of Th17, favoring instead the differentiation of Foxp3^+^ Treg cells (Berod et al., 2014). Consistent with these results, we have recently shown that in the presence of lactate, at concentrations comparable to those measured in the synovial tissue, CD4^+^ T cells upregulate the *de novo* FAS, leading to increased IL-17 and reduced cell motility (Pucino et al., 2019). Interestingly, these events were restored after treating CD4^+^ T cells with a range of FAS inhibitors and reducing the lactate-induced NADPH levels (Pucino et al., 2019).

While *de novo* FAS has been shown to play an important role in effector CD4^+^ T cell functions, cholesterol metabolism is involved in the regulation of the anti-inflammatory response in human CD4^+^ T cells (Perucha et al., 2019). Inhibition of the cholesterol biosynthesis pathway with atorvastatin or 25-hydroxycholesterol during switching from IFNγ^+^ to IL-10^+^ showed a specific block in immune resolution, defined as a significant decrease in c-Maf/IL-10 expression (Perucha et al., 2019).

Metabolomics profiling has shown alterations in the lipid metabolism in RA versus OA FLS. In line with this evidence, choline and choline like transporter (CTL)1 (high-affinity) and CTL2 (low affinity), were found to be highly expressed by synovial RA FLS (Ahn et al., 2016; Volchenkov et al., 2017) and their functional inhibition promoted FLS cell death (Seki et al., 2017). Supporting these findings, positron emission tomography (PET) scanning with ^11^C-choline showed increased uptake in inflamed arthritic joints (Seki et al., 2017). Further studies are needed to understand the mechanisms linking lipid metabolism to FLS effector functions and subset differentiation in RA.

## Tanscription Factors as Metabolic Sensors

Catabolic and anabolic pathways are regulated by specific transcription factors which act as metabolic sensors. In this context, 5′ AMPK is a redox sensor, being activated by increased AMP:ATP ratios (Shirwany and Zou, 2014). AMPK modulates several metabolic functions, including glucose uptake, mitochondrial biogenesis and lipid metabolism, as well as cellular functions (i.e., transcriptional activity and cell cycle). Therapeutic AMPK activation was reported to suppress experimental arthritis. Moreover, methotrexate-induced activation of AMPK-dependent pathway has been shown to protect the vasculature against inflammation (Kang et al., 2013; Yan et al., 2015; Thornton et al., 2016). AMPK activation is myristoylation dependent. Notably, RA T cells display a defect in N-myristoyltransferase (NMT) function, which prevents AMPK activation and enables mTORC1 signaling activation, resulting in pro-inflammatory Th1 and Th17 differentiation. NMT1 loss of function experiments induced an inflammatory response both *in vitro* and *in vivo*; by contrast, NMT1 overexpression restored AMPK activation and suppressed synovial inflammation (Wen et al., 2019).

Finally, metformin, an anti-diabetic drug, which indirectly activates AMPK, has been shown to mitigate disease in mouse models of arthritis (Son et al., 2014) *via* the inhibition of mTOR pathway, the suppression of NF-κB-mediated inflammatory cytokine production as well as enhanced autophagic flux (Yan et al., 2015).

Together with AMPK, mTOR is a central integrator of environmental signals and nutrient availability with cellular functions (Delgoffe and Powell, 2015; Pollizzi and Powell, 2015; Pucino et al., 2016). Indeed, aberrant mTOR activation is associated with cellular senescence, and rapamacyin, the mTOR complex 1 inhibitor, has been investigated as a therapeutic agent to treat degenerative, autoimmune and hyperproliferative diseases (Perl, 2016). The ability of mTOR to integrate nutrient supply, bioenergetics and T cell functions, makes it a promising target for therapeutic intervention to suppress abnormal T cell differentiation during the early stages of RA (Perl, 2016).

## Adipo-Cytokines

A link between the neuroendocrine and immune systems has been shown to contribute to the pathogenesis of several immune mediated inflammatory disorders (Cassano et al., 2014; Procaccini et al., 2014). In this context adipo-cytokines, such as leptin and adiponectin, hormones secreted mainly by the adipose tissue, have been shown to play a role in RA pathogenesis (Hamaguchi et al., 2012; Ruscitti et al., 2018). For instance, it has been shown that *ob/ob* mice develop resistance to experimental antigen-induced arthritis compared to wild-type mice (Busso et al., 2002). In addition a decrease in serum leptin concentration following fasting, limited CD4^+^ activation, promoted a shift toward Th2-type cytokine secretion, and improved clinical disease in RA patients (Fraser et al., 1999). Leptin can also directly modulate chondrocyte biology. Indeed, leptin induces, in combination with IFN-γ and IL-1, nitric oxide synthases (NOS) type II activation in cultured chondrocytes (Otero et al., 2003). These events promote pro-inflammatory cytokine production in joint cartilage, causing chondrocyte apoptosis, metalloproteases activation and consequently inflammation (Otero et al., 2005). However there is conflicting evidence regarding the role of leptin in RA (Tian et al., 2014). Some studies have found elevated leptin in serum from RA patients (Bokarewa et al., 2003; Xibille-Friedmann et al., 2010; Yoshino et al., 2011) in particular in patients with erosive RA (Targonska-Stepniak et al., 2010; Olama et al., 2012). Conversely other reports have showed no difference in serum leptin levels between RA patients and healthy controls (Harle et al., 2006; Hizmetli et al., 2007; Oner et al., 2015). Leptin has also been detected in RA synovial fluid and tissue. A study by Seven et al. (2009) reported that serum and synovial fluid leptin levels were higher in RA patients when compared to controls, with positive correlation with disease activity. Another study showed instead a negative correlation between leptin synovial fluid levels and bone erosions. In addition, leptin levels were higher in the serum than in the synovial fluid suggesting that leptin may be consumed in the joints and have a protective role against erosions (Bokarewa et al., 2003).

Similar to leptin, adiponectin has also been suggested to play a role in the pathogenesis of RA, though again results are inconsistent. Adiponectin is a 28–30 kDa collagen-like protein predominantly secreted by adipocytes. In some studies, increased levels of adiponectin were found in synovial fluid and serum of patients with RA (Schaffler et al., 2003; Otero et al., 2006) and were associated with the production of pro-inflammatory mediators and arthritis (Ehling et al., 2006). In other studies, serum adiponectin showed no association or a negative correlation with disease activity in RA (Senolt et al., 2006; Rho et al., 2009; Yoshino et al., 2011). In the DBA/1 mouse model of collagen-induced arthritis, adiponectin treatment significantly alleviated the severity of arthritis together with a decrease in the expression of pro-inflammatory cytokines such as TNF-α and IL-1, and the reduction of metalloproteinase (MMP)-3 in synovial tissues (Lee et al., 2008). These latter findings suggest that in RA the role of adiponectin is anti-inflammatory rather than pro-inflammatory.

## Treatments Affecting Metabolic Signaling Pathways in RA

Several drugs currently in use to treat RA affect metabolic signaling pathways. Glucocorticoids for example, inhibit the glycolytic enzyme fructose 2,6-bisphosphate in rat tymocytes and regulate respiratory rate in peripheral blood mononuclear cells from patients with rheumatic diseases (Moreno-Aurioles and Sobrino, 1991; Kuhnke et al., 2003). Methotrexate’s anti-inflammatory effects depend on the modulation of purine or pyrimidine nucleotide metabolism (Cronstein and Aune, 2020). Similarly, biologic disease modifying anti rheumatic drugs (DMARDs) can modulate specific metabolic pathways. For example, anti-TNF-α and JAK inhibitor (i.e. tofacitinib) treatments decrease glycolysis in RA synovium (Biniecka et al., 2016; McGarry et al., 2018).

In the context of tofacitinib, it significantly increased oxidative phosphorylation, mitochondrial respiration in RA FLS, coupled with a decrease in glycolysis and several key glycolytic enzymes such as HK2, glycogen synthase kinase 3α (GSK-3α), lactate dehydrogenase A, and HIF-1α both in RA FLS and synovial explants (McGarry et al., 2018). It would be interesting to evaluate if these events are associated with reduced lactate levels and impaired lactate/STAT3 signaling as we have recently shown (Pucino et al., 2019).

The anti IL-6 receptor antibody tocilizumab decreases oxidative stress in RA leucocytes (Ruiz-Limon et al., 2017). Over-expression of HK2 has been associated with resistance to rituximab (anti-CD20) in aggressive lymphoma, whilst the impact of rituximab on immune cell metabolism in RA patients is still unknown (Gu et al., 2018).

## Conclusion and Further Perspectives

The tissue microenvironment plays a pivotal role in the pathology of inflammatory diseases such as RA. A lack of nutrients, low oxygen concentrations, accumulation of metabolic intermediates as well as unbalanced metabolic pathways drive the local immune response in such a way as to exacerbate chronic inflammation (Figure 1).

Immunometabolism studies have recently highlighted the possibilty of targeting metabolic pathways, metabolites, transcription factors and enzymes that are altered in RA (Figure 1 and Table 1). Several drugs currently in use to treat RA affect metabolic signaling pathways. However, we are now in a position from which we can consider developing therapies to specifically target pathogenetically relevant metabolic pathways. For example, targeting specific metabolic pathways has been demonstrated to reduce inflammation both *in vitro* and *in vivo* models of arthritis (Yan et al., 2015; Biniecka et al., 2016; Okano et al., 2017; Shen et al., 2017; Bustamante et al., 2018). In addition, targeting metabolic intermediates such as lactate (Pucino et al., 2018; Certo et al., 2019) or succinate (Littlewood-Evans et al., 2016), is also becoming an attractive possibility. Animal models remain a crucial tool for preclinical screening of new therapeutics in pharmaceutical development. However, potential therapeutics, which have been shown to be safe and effective in animal studies, have in certain cases failed when tested in humans. Further knowledge on human immunology and additional development of animal models that bear more resemblance to the human condition are needed (Hegen et al., 2008; Bevaart et al., 2010). Another important area of investigation is the impact of sex and gender on RA immunometabolism. Prevalence of RA is higher in women than in men (van Vollenhoven, 2009). This is partly ascribed to the effect of sex hormones on the immune system and their interaction with environmental and genetic factors (Alpizar-Rodriguez et al., 2017). Estrogenic control of mitochondrial function and glycolysis metabolism has been studied (Cai et al., 2013; Klinge, 2020), however what are the sex-based differences in RA cell immunometabolism is still unknown and needs further investigation.

**TABLE 1:** Potential metabolic therapeutic targets in RA.

Cells	Defective metabolic Pathway	Potential therapeutic targets
T cell	Glycolysis (−) PPP (+) Lipid (+)	PFKFB3 G6PD FASN Lactate/SLC5A12 AMPK/mTOR
Monocytes/macrophages	Glycolysis (+) TCA (+) AMPK (−)	PKM2 HIF Succinate/GPR91 Lactate/MCT1 and 4
Fibroblasts	Glycolysis (+) Lipid (+)	GLUT1 HK2 PFKFB3 Choline/Chokα
Dendritic cells	Glycolysis (+)	HK2 iNOS AKT/mTOR

*AMPK, adenosine monophosphate-activated protein kinase; Chokα, choline kinase alpha; FAS, fatty acid synthase; G6PD, glucose-6-phosphate dehydrogenase; G6PI, glucose 6 phosphate isomerase; HK2, hexokinase 2; iNOS, inducible nitric oxide synthase; HIF-1α, hypoxia-inducible factor 1-alpha; MCT, monocarboxylate transporter; mTOR, mammalian target of rapamycin; PFKFB3, 6-phosphofructo-2-kinase/fructose-2, 6-bisphosphatase; PKM2, pyruvate kinase muscle isozyme 2; PPP, pentose phosphate pathway; SLC5A12, solute carrier 5A12; (−) decreased, (+) enhanced.*

Correlation studies between serum metabolites and synovial and blood biomarkers suggests that NMR and mass-spectrometry may be promising tools for predicting specific pathogenic pathways altered in RA (Young et al., 2013; Zhou et al., 2016; Narasimhan et al., 2018). In addition they may be useful in the future to to identify which RA patients are at higher risk to develop artheroslerosis. Metabolomics profiles in serum, plasma, or urine do not necessarily correlate with joint metabolism as well as synovial fluid metabolites may not identify metabolic pathway alterations in the synovial tissues.

Further studies are needed to better determine whether specific metabolic signatures can be used to stratify patients with RA in terms of outcome, disease stage and response to therapy. Single cell RNA-seq techniques will be of help to shed light on metabolic pathways used by specific immune cells (i.e., macrophages, lymphocytes, fibroblast) in the context of the RA inflammatory environment.

Advanced RNA-seq techniques are also developing. In this context, the droplet-based single-cell RNA-seq has recently been shown to be a promising tool for cellular profiling allowing the analysis of thousands of individual cells simultaneously by encapsulating them in tiny droplets (Salomon et al., 2019). Similarly single cell metabolomic analysis will facilitate the identification of new biomarkers and the development of novel therapeutic molecules targeting abnormal metabolic signaling pathways at single cell level without dampening homeostatic immune responses.

## Author Contributions

VP, CM, KR, and CB contributed to the conceptualization. VP, MC, CM, KR, and CB contributed to the preparation of the original draft. VP, MC, GV, GM, FU, FR, AD, CM, KR, and CB contributed to the final editing and revision.

## Conflict of Interest

The authors declare that the research was conducted in the absence of any commercial or financial relationships that could be construed as a potential conflict of interest.
